# Effect of auricular electroacupuncture combined with body acupuncture in improving the consciousness of patients after traumatic brain injury: Study protocol for a randomized controlled trial

**DOI:** 10.1097/MD.0000000000016587

**Published:** 2019-07-26

**Authors:** Tong Liu, Yanqing Lu, Jiani Yu, Weichuan Kuang, Xiaoyin Wang, Ye Jiang, Xiaojia Qiu, Xi Wen, Yao Zeng, Guitao Zhang, Yue Liu

**Affiliations:** a Department of Acupuncture and Rehabilitation, GuangDong Second Hospital of Traditional Chinese Medicine; b Department of Acupuncture and Rehabilitation, Guangzhou University of Chinese Medicine; c Department of Rehabilitation Medicine, Guangdong Provincial Hospital of Traditional Chinese Medicine, Guangzhou, Guangdong, China.

**Keywords:** auricular acupoint, body acupuncture, consciousness, electro-acupuncture, GCS, mismatch negativity, traumatic brain injury

## Abstract

**Background:**

Traumatic brain injury (TBI) has become a major cause of morbidity and mortality both in China and abroad. Disorders of consciousness following severe TBI is a common refractory complication, resulting in difficult rehabilitation and poor life quality. However, effective therapeutic approaches remain limited. Although acupuncture has been widely applied in the treatment of neurological disorders in China, its efficacy and safety for consciousness recovery remain to be elucidated.

**Methods:**

Here, we conduct a study design and protocol of a randomized, blinded, controlled study to evaluate the efficacy and safety of electroacupuncture at auricular acupoints “heart” and “brainstem” combined with body acupuncture in the consciousness recovery of patients with TBI. A total of 80 patients with initial Glasgow coma scale score between 3 and 8 points will be recruited in the trial and randomized into intervention (combined application of auricular electroacupuncture and body acupuncture) group or control (conventional treatment) group. Patients in the intervention group will receive electroacupuncture at bilateral auricular acupoints “heart” and “brainstem” (4 points in total) combined with body acupuncture in addition to conventional treatment while patients in the control group will receive conventional treatment alone for 8 weeks. The primary outcomes are changes of Glasgow coma scale score and mismatch negativity of event-related brain potentials at baseline after 4 weeks after the final treatment and 4 weeks after the final treatment. The secondary outcome measures will be changes of Barthel and FuglMeyer scores at baseline after 4 weeks after the final treatment and 4 weeks after the final treatment. The safety will also be assessed by monitoring the incidence of adverse events and changes in vital signs during the study.

**Discussion:**

Results from this trial will significantly support the application of auricular acupuncture and body acupuncture in the consciousness recovery of patients with severe TBI. If found to be effective and safe, auricular acupuncture combined with body acupuncture will be a valuable complementary option for comatose patients with TBI.

**Trial registration:**

Chinese Clinical Trial Registry: ChiCTR1800020245. Registered on 21 December 2018.

## 1 Introduction

Traumatic brain injury (TBI) is a leading cause of morbidity and mortality among children and young adults.^[1]^ In China, it is reported that the mortality rate induced by TBI was among 2.7% to 21.8%.^[2]^ Although the number of patients survived from severe TBI increased gradually accompanied by the advance in neurocritical care, many survivors still failed to fully recover a state of disorders of consciousness (DOC) such as coma, vegetative state, and minimally conscious state.^[3]^ The neuropathology of DOC has been extensively described at postmortem and diffuse disruption of subcortical white matter was reported the most common reasons found in victims of TBI induced DOC.^[4,5]^ Longer time to recovery from DOC is predictive of worse prognosis after TBI,^[6,7]^ and shortening the period of DOC may be helpful in improving outcomes, at least partly increase participation in rehabilitation treatment.^[8,9]^ However, only few limited strategies have been found for the treatment of DOC.

As a complementary and alternative therapy, acupuncture is an important component of traditional Chinese medicine and has been widely used in various diseases during the past thousands of years in China. Many types are involved in acupuncture treatment, such as body acupuncture, auricular acupuncture, scalp acupuncture, wrist-ankle acupuncture, and so on. Body acupuncture has been proved in improving consciousness induced by TBI in many studies.^[10,11]^ Auricular acupuncture has also been accepted in many disorders or conditions, such as pain,^[12]^ constipation,^[13]^ addiction,^[14]^ insomnia,^[15]^ cognitive impairment, and dementia^[16]^; however, there combined effect for DOC has not been examined.

Thus, we present a study design and protocol of a randomized, blinded, controlled study to assess the efficacy and safety of auricular electroacupuncture combined with body acupuncture in improving the consciousness in patients with TBI. The objective of this study was to determine if auricular electroacupuncture combined with body acupuncture is more effective than conventional treatment. The results of this study are expected to establish an optimal acupuncture procedure for DOC.

## 2 Methods

### 2.1 Objective

In this study, the efficacy of combined application of auricular and body acupuncture for consciousness recovery of patients with TBI versus conventional treatment will be evaluated.

### 2.2 Trial design and setting

**Figure 1:**
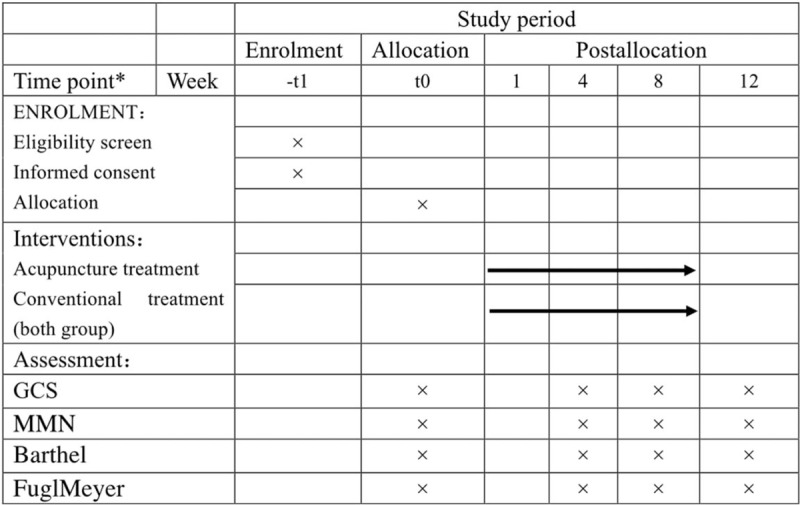
Schedule of enrollment, interventions, and assessments.

This study is a single-center, randomized controlled, assessor-blinded clinical trial which was devised following the consolidated standards of reporting trials Statement recommendations.^[17]^ The total study period for this trial is 12 weeks (Fig. 1), including 8-week treatment phase and 4-week follow-up phase.

**Figure 2:**
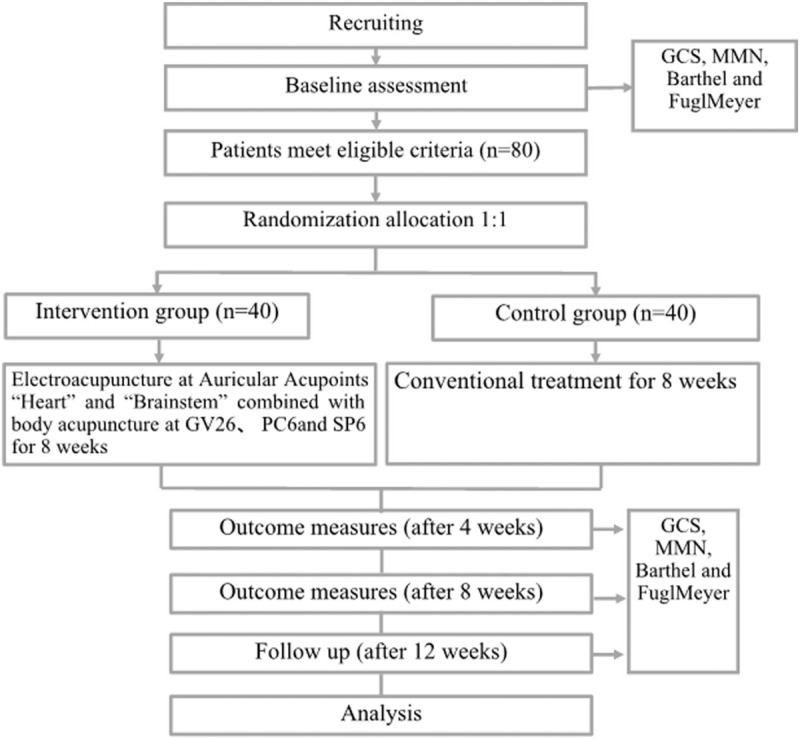
Flow chart of the study procedure.

It will be performed in the GuangDong Second Hospital of Traditional Chinese Medicine. 80 patients who meet the eligibility criteria and sign an informed consent form will be randomly divided into 2 groups to receive either auricular electroacupuncture plus body acupuncture or conventional treatment in a 1:1 ratio. Acupuncture will be performed 30 minutes per day, 5 days per week for 8 weeks. Doctors with more than 5 years of clinical experience will be allowed to perform the interventions. The flowchart of the trial is shown in Figure 2.

### 2.3 Participants

#### 2.3.1 Recruitment strategies

Inpatients at acupuncture departments in GuangDong Second Hospital of Traditional Chinese Medicine will be recruited mainly in this randomized controlled trials. Additionally, posters will be put in the hospitals and a Chinese multipurpose social media named WeChat will be applied to recruit. Brief descriptions of eligible criteria, the free acupuncture treatments and the possible risks of the trial will be marked and all patients’ family members have the right to participate or drop out at any time, and will be required to sign the informed consent before the trial begin.

#### 2.3.2 Eligibility criteria: inclusion criteria

The inclusion criteria will be as follows.

1. Meet the diagnostic criteria for TBI;
2. Aged 18 to 70 years old, male or female;
3. Initial GCS score between 3 and 8 points on admission and lasted more than 2 weeks;
4. Imaging (magnetic resonance imaging) head has no obvious shift, missing, large necrosis of brain structure change and obvious brain stem (not including pyramidal tract) or thalamic lesions, each lobe lesions range can not exceed 30% of the scope of 1 side of the brain;
5. No primary consciousness disorder and limb functional activity disorder;
6. Signed informed consent.

#### 2.3.3 Eligibility criteria: exclusion criteria

The exclusion criteria will be as follows.

1. DOC not induced by TBI;
2. Clinical course more than 1 year;
3. Combination of heart, liver or kidney failure endanger the safety of life at any time;
4. Younger than 18 years or older than 70 years;
5. Those who do not receive electroacupuncture therapy;
6. Women with pregnancy and lactation.

### 2.4 Randomization and allocation

Random numbers will be generated by the random number generator in the SPSS statistical software package (version 20.0, IBM SPSS Statistics, IBM Corp, Somers, NY). Another specified researcher who is not involved in the study was responsible for it.

The allocation of participants will be sealed in a sequentially numbered opaque envelope. If the participant meets the inclusion criteria and signs informed consent, the above-specified researcher will sequentially give the sealed random number envelope to the physician, who will open the envelope and allocate the participant to either intervention group or control group according to the random number.

### 2.5 Blinding

Because of the add-on study design, a single-blinded method will be used. While the participants and practitioners cannot be blinded, we blinded the outcome assessors, data manager, and statistics analyzer. The assessor will be instructed not to communicate with participants about the possibility of their treatment. James et al's blinding index will be evaluated after the completion of the study to evaluate the success of blinding.^[18]^

### 2.6 Intervention

In the 8-week treatment phase, participants in both groups will receive conventional treatment, and those allocated to the intervention group will also receive auricular electroacupuncture combined with body acupuncture treatment.

### 2.7 Conventional treatment in both groups

All the participants will accept conventional treatment, including the prescription of coma arousal and neuroprotective medicines. Details of conventional treatment used during the trial will be recorded, noting any change and the reasons.

Auricular electroacupuncture combined with body acupuncture in the intervention group.

In addition to conventional treatment, patients in the intervention group will receive both auricular point electroacupuncture and body acpuncture. Auricular point electroacupuncture manipulation was as follows: Following disinfection of skin with 75% alcohol, bilateral auricular acupoints “heart” and “brainstem”^[19]^ (4 points in total) will be stimulated using disposable and stainless needles (13 mm long and 0.25 mm in diameter, Suzhou Huanqiu Acupuncture Medical Appliance, Suzhou, China) inserted to a depth of 2 mm, and connected to an electrical stimulator (SDZ-V EA; Huatuo, Suzhou, China) with a frequency of 2/10 Hz and current of 1 mA. Body acupuncture manipulation was as follows: After disinfection, GV26^[20]^ was inserted towards the nose at a depth of 10 mm while bilateral PC6^[20]^ and SP6^[20]^ was perpendicularly inserted at a depth of 20 mm by disposable and stainless needles (40 mm long and 0.30 mm in diameter, Suzhou Huanqiu Acupuncture Medical Appliance). The needle will be manipulated using twirling, lifting, thrusting, and mild reinforcing-reducing methods to promote Qi. Eye moisture or the presence of tears will be used as an indicator of Qi arrival since patients are not able to express their feelings. Acupuncture will be restrained for 30 minutes, once daily, 5 times a week and lasted for 8 weeks. The location of acupoints applied was shown in Table 1.

**Table 1:**
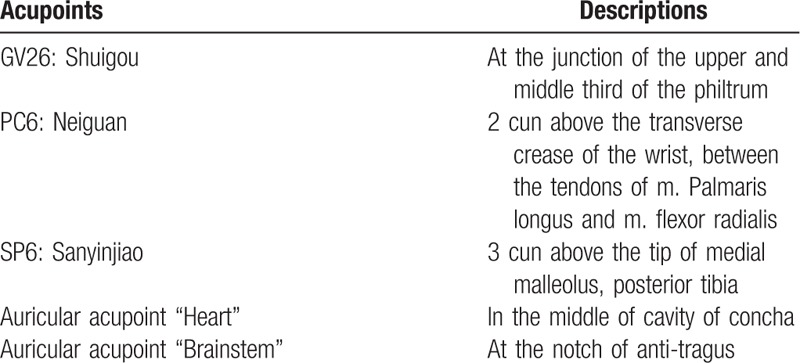
Description of the applied auricular points.

### 2.8 Discontinuing interventions

The trial will be ceased if one of the following conditions appeared:

(1) a serious poststroke complication arises; or
(2) recurrent stroke or any other severe condition occurs leaving the patient in a critical condition.

### 2.9 Outcome measures

The primary efficacy endpoint will be the Glasgow Coma scale (GCS) and mismatch negativity (MMN) at baseline after 4 weeks after the final treatment and 4 weeks after the final treatment. The secondary outcome measures will be changes of Barthel and FuglMeyer scores at baseline after 4 weeks after the final treatment and 4 weeks after the final treatment. Safety will be assessed by monitoring adverse events as well as changes in vital signs during the study.

### 2.10 Safety assessment

Any adverse events, including acupoint hematoma, infection, dizziness, and apostasies will be evaluated during the whole treatment by the researcher. If any severe adverse events occurring, acupuncture intervention will be ceased immediately and the principal investigator will be informed to take proper actions. 1 month will be followed up after the trial in case of subsequent adverse events.

### 2.11 Data collection

All the study data will be recorded on the case report form (CRF) and any corrections made to the CRF must be personally signed and dated by the person responsible. All data will then be entered into a predesigned, password-protected electronic data set by 2 independent investigators who are blinded to group allocation. Double-checking of entered data will be performed by another researcher to ensure accuracy.

### 2.12 Sample size calculation

The trial is designed to determine the role of auricular and body acupuncture in the consciousness recovery of patients with TBI and prove that auricular and body acupuncture is superior to conventional treatment. Therefore, GCS change will be used as an evaluation index. According to our previous pilot study, the change of GCS before and after treatment in the intervention group was shown to be 5.62 ± 1.41 (n = 8) and that in control group was 4.50 ± 1.85 (n = 8). Sample size was estimated using the following formula: 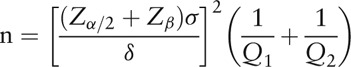


where n represents the number of samples required, n = n_1_ + n_2_, *Q*_1_ = n_1_/n, Q_2_ = n_2_/n with a significance level (*α* = 0.05) of a 2-sided 2-sample *t* test and 80% power to detect a difference between the 2 groups. Thus, a total sample size of 80 patients will be recruited allowing for 10% of attrition, with 40 in each.

### 2.13 Statistical analysis

SPSS 20.0 (IBM SPSS Statistics, IBM Corp, Somers, NY) will be used to analyze the data. Quantitative data will be presented as mean ± standard deviation. GCS Barthel and FuglMeyer scores will be conducted between the 2 groups by a superiority independent sample *t* test with a 95% confidence interval. The incidence of complications and adverse events will be compared by *χ*^2^ test. A mixed model procedure will be applied to compare longitudinal changes in MMN amplitude between the 2 groups. Any missing data will be replaced by the last measured value. For all analyses, *P* values of less than .05 will be considered statistically significant.

### 2.14 Quality control

Before the trial, all the acupuncturists nurses and assessors will be trained strictly in order to guarantee homogeneity in the measurement data and ensure high-quality data results. The training content will include study protocol, diagnosis, inclusion and exclusion criteria, recording method of CRF, the location of the acupoints, acupuncture operation techniques, disposal of bleeding. All the study data will be recorded on the CRF. Dropouts and withdrawals from the study will be recorded in detail based on the intervention and follow-up periods. Data will be uploaded and verified by other 2 researchers who were not involved in the trial. This trial will be monitored by the Scientific Research Department of GuangDong Second Hospital of Traditional Chinese Medicine every 1 week.

### 2.15 Ethics and dissemination

We strictly follow the principles of the medical ethics of the Declaration of Helsinki^[21]^ with the approval of the Ethics and Research Committee of GuangDong Second Hospital of Traditional Chinese Medicine, China. If there are any protocol modifications, we will report to the Ethics and Research Committee for approval. All patients will be recruited from the Department of Acupuncture and Rehabilitation, GuangDong Second Hospital of Traditional Chinese Medicine. Informed consent will be obtained from all study participants. Participant information will be protected. All experimental data will be stored in a secure storage area with access limited to the researchers alone. We will disseminate the results of this study in meetings or publications when the trial is completed.

## 3 Discussion

The incidence of TBI has become more common with the rapid societal development and increase in traffic accidents.^[22,23]^ It affects at least 1.7 million individuals in the United States and causing 1.5 million hospitalizations in the European Union yearly.^[24–26]^ It is also a serious public health problem in China and causes a substantial number of deaths and temporary and permanent disabilities.^[27]^ Even if survives, many patients will fall into a long period of DOC. Thus, it is urgent to found more effective and safe strategies to improve the outcome of patients with DOC.

Acupuncture has been widely applied in China and abroad to treat neurological diseases, its effect for DOC has also been certified by some studies.^[28,29]^ Here, we conducted a study to compare the effect of auricular combined with body acupuncture to conventional treatment in order to clarify if auricular combined with body acupuncture could strengthen the awaking role. In our trial, strict quality control procedures have been applied to avoid bias, such as randomization and allocation concealment, assessor blinding and adequate sample size. However, methodological limitations still exist. Due to the nature of acupuncture manipulation, the therapists and the participants cannot be blinded in this study.

In this study, the acupuncture points of PC6 GV26 and SP6 were selected based on Shixuemin's Xingnaokaiqiao theory.^[30]^ Xingnaokaiqiao method has been applied by many clinical doctors to treat mental diseases and reported satisfactory clinical curative effects.^[31,32]^ PC6 is an acupoint mainly used to treat mind-mental disorders and heart diseases in traditional Chinese medicine. It has been proved that PC6 acupuncture could increase connections between cerebral cortex regions.^[33]^ GV26 also plays a positive role in recovery of neurological function after stroke. Animal research has indicated that electroacupuncture at GV26 improves neurological function in rats following cerebral ischemia/reperfusion injury, and may be associated with downregulation of BKCa channel protein and mRNA expression.^[34]^ Functional magnetic resonance imaging studies revealed that acupuncture at SP6 could induce changes in neural activity in the sensorimotor cortical network of a sleep-deprived brain.^[35]^ Auricular acupuncture (AA) is a safe nonpharmaceutical complementary medicine technique based on a hypothesis that the entire human body is represented on the external auricle.^[36]^ The superiority of AA has been examined in many disorders, such as pain,^[37]^ constipation,^[38]^ insomnia^[39]^ and congnitive impairment.^[40]^ The auricular point “heart” is located in the middle of the ear armor cavity and is closely related with consciousness according to the traditional Chinese theory “Heart Govern the Spirit Light.” Previous studies have certified that stimulation at auricular point “heart” and “brainstem” could have positive effect on brain function.^[41–43]^ It is worth mentioning that the position of the 2 points are partially coincident with the distribution of the auricular vagal nerve, which is considered to be a mechanism behind the analgesic effects of auricular acupuncture.^[44,45]^

In conclusion, this study will provide solid evidence of the role of acupuncture in improving the consciousness of patients after TBI.

### 3.1 Trial status

This is protocol version1.0, version date is 2018.9.19. Participants are being recruited. Recruitment began on 1 June 2018. The trial is planned to be completed by 30 June 2020.

## Acknowledgments

We are thankful to Prof. Nie Bin, GuangDong Second Hospital of Traditional Chinese Medicine, for scientific designment.

## Author contributions

**Data curation:** Weichuan Kuang, Xiaoyin Wang.

**Formal analysis:** Ye Jiang.

**Project administration:** Tong Liu, Xiaojia Qiu, Xi Wen, Yao Zeng, Guitao Zhang.

**Resources:** Yue Liu.

**Supervision:** Yue Liu.

**Writing – original draft:** Tong Liu, Yanqing Lu.

**Writing – review and editing:** Yanqing Lu, Jiani Yu.
